# Supplementary material for: Conditional chemoconnectomics (cCCTomics) as a strategy for efficient and conditional targeting of chemical transmission
Source: eLife. 2024 Apr 30;12:RP91927. doi: 10.7554/eLife.91927 (PMC11060718; doi:10.7554/eLife.91927)
Supplement: Figure 3—source data 3. [file elife-91927-fig3-data3.zip › Figure 3 source data3 part 2.zip!/Figure 3-Source data 3-part2/1060-9-CRISPResso_Report_q0_g4o36.zip!/CRISPResso_on_1060_10/1c.Alignment_barplot.pdf]

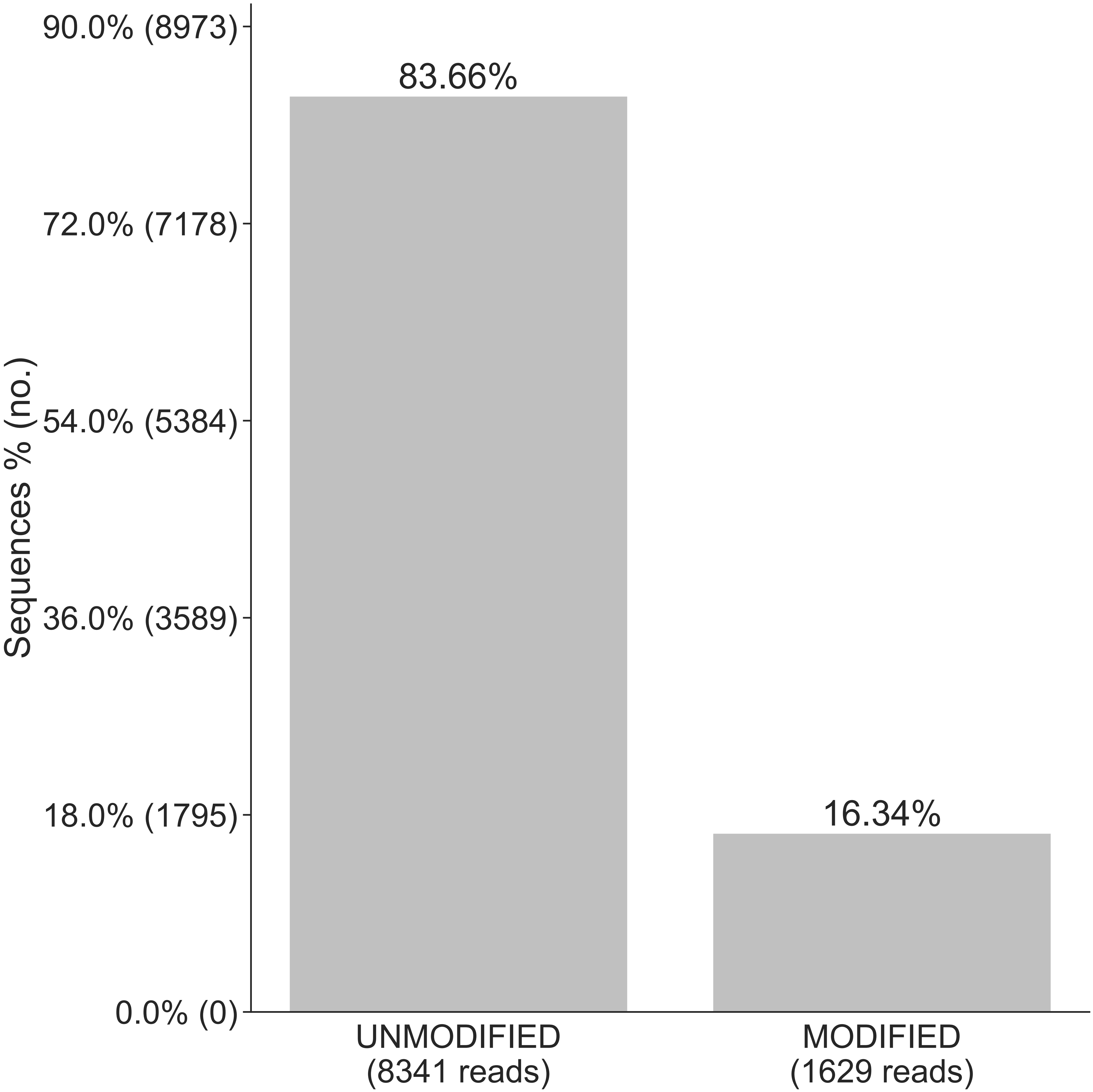

Sequences % (no.)
90.0% (8973)
72.0% (7178)
54.0% (5384)
36.0% (3589)
18.0% (1795)
0.0% (0)
83.66%
16.34%
UNMODIFIED
(8341 reads)
MODIFIED
(1629 reads)
